# Appropriate Nitrogen Addition Boosts Coastal Wetland Carbon Sequestration: *Kandelia obovata* Optimizes Microbial Carbon Use Strategies

**DOI:** 10.3390/plants15101470

**Published:** 2026-05-12

**Authors:** Huiming You, Wanlong Ni, Jiangrong Lv, Fanglin Tan, Xiaoxue Yu, Jianliang Han, Weibin You

**Affiliations:** 1 Fujian Academy of Forestry, Fuzhou 350012, China; youhuiming@126.com (H.Y.); hjlsdau@163.com (J.H.); 2 Wetland Ecosystem Research Station in Quanzhou Estuary, Quanzhou 362000, China; 3 College of Forestry, Fujian Agriculture and Forestry University, Fuzhou 350002, China; niwanlong0705@163.com (W.N.); lvjron@163.com (J.L.); htqlyj@163.com (X.Y.); 4 Research Centre of Southern Forest Resources and Environment Engineering Technology of Fujian Province, Fuzhou 350002, China

**Keywords:** nitrogen deposition, coastal wetland ecosystems, carbon sequestration, microbial metabolic strategies, Biolog-ECO

## Abstract

Mangrove ecosystems in coastal wetland restoration areas are experiencing escalating nitrogen stress, yet the microbial metabolic mechanisms underlying soil carbon sequestration in *Kandelia obovata* systems under exogenous nitrogen input remain unclear. In this laboratory tidal simulation experiment, five nitrogen addition levels (N0–N4) were applied to two treatments, namely the planted group and the unplanted group. Results showed that total carbon (TC), microbial biomass carbon (MBC), and microbial biomass nitrogen (MBN) were all higher under nitrogen addition than in the N0 control. TC showed a unimodal response to nitrogen addition, with the highest values observed at N2, while the planted group exhibited the greatest relative increase in TC over the unplanted group at N3 (53.49%). MBC and MBN contents initially increased and then decreased with elevated nitrogen addition, peaking at the N3 treatment. Compared with the N0 control, MBC and MBN contents under N3 increased by 31.83% and 206.24% in the planted group, and by 23.46% and 279.03% in the unplanted group, respectively. Microbial carbon source utilization was stronger in the planted group, where microorganisms preferred amino acid and lipid carbon sources. Microbial communities in the unplanted group fluctuated markedly under nitrogen input, whereas those in the planted group were more stable with higher evenness. In the planted group, nitrogen addition promoted carbon sequestration by enhancing microbial activity and biomass accumulation, while in the unplanted group, nitrogen input exerted complex effects and directly suppressed soil carbon sequestration. These findings suggest that the introduction of *Kandelia obovata* may enhance microbial biomass, stabilize microbial carbon-use strategies, and promote short-term soil carbon accumulation under moderate nitrogen addition in a laboratory tidal simulation system. Overall, the N3 treatment (20 g N m^−2^ a^−1^) serves as a key nitrogen threshold, and exceeding this addition level may weaken the beneficial effects on microbial biomass, metabolic activity, and the relative carbon accumulation advantage of the planting system.

## 1. Introduction

Mangroves serve as important carriers for coastal ecological restoration and key contributors to blue carbon sequestration. They regulate the global carbon cycle through processes such as organic carbon accumulation and methane emission, and play a pivotal role in maintaining ecosystem stability and mitigating climate change [1]. Since the 1970s, mangroves have become the preferred afforestation type for global coastal wetland ecological restoration, with numerous countries successively launching mangrove restoration initiatives. In China, continuous improvements in mangrove conservation and restoration policies have further promoted their large-scale application in estuarine and intertidal wetlands [2,3]. Following the eradication of invasive *Spartina alterniflora* in Fujian, *Kandelia obovata* has emerged as a dominant restored mangrove species, signifying its key function in sustaining wetland integrity and blue carbon sink capacity [4]. In recent years, the extensive use of fossil fuels and chemical fertilizers in production and daily life has accelerated the input of substantial nitrogen into coastal wetland ecosystems. Previous studies have indicated that nitrogen addition can also alter the diversity and composition of soil microbial communities in mangroves, thereby affecting mangrove carbon sequestration efficiency [5]. It is projected that global nitrogen deposition will reach 195 Tg·a^−1^ by 2050 [6]. Nitrogen is a key limiting nutrient in mangrove wetland ecosystems [7,8]: appropriate nitrogen addition can promote mangrove growth and enhance its carbon sequestration capacity, while excessive nitrogen addition may cause damage to mangrove roots [9]. When plant biomass increases, the carbon storage capacity of soil may conversely decrease; this trade-off relationship may reduce soil carbon sequestration capacity [10].

Soil microbial communities are critical components of mangrove ecosystems, which can sensitively reflect changes in the ecological environment and predict the dynamic trends of wetland ecosystems under nitrogen deposition [11,12]. Different nitrogen input concentrations exert varying impacts on soil carbon–nitrogen cycles and soil microbial carbon source utilization efficiency [13]. Currently, there are significant international debates regarding the “nitrogen-promoted carbon sink” effect, with findings supporting increased, decreased, or unchanged carbon sink capacity [14,15]. Duan et al. [16] (2025) identified essential differences in the microbial mechanisms of carbon sequestration between *Sonneratia caseolaris* and *Kandelia obovata*: in *Sonneratia caseolaris*, more r-strategist microorganisms rapidly convert plant-derived carbon into biomass and residues, leading to a positive correlation between recalcitrant organic carbon and amino sugars; in *Kandelia obovata*, K-strategist fungi in the surface soil enhance enzyme activity, while K-strategist bacteria in the subsoil strengthen carbon utilization capacity, jointly facilitating the conversion of plant residues into lignin phenols and lipids, with a stronger correlation between recalcitrant organic carbon and the latter two. You [17] (2023) also found that exogenous nitrogen addition had no significant effect on plant carbon and nitrogen content, but an extremely significant effect on soil microbial biomass carbon and nitrogen. The presence of mangrove plants promoted soil microbial activity: low and medium nitrogen concentrations facilitated the accumulation of soil microbial biomass carbon and nitrogen, whereas high nitrogen concentrations exerted an inhibitory effect. Zhu et al. [18] (2024) investigated the spatial distribution of carbon metabolic activity and carbon source utilization preferences of soil microorganisms in saline-alkali wetlands, and found that microbial carbon metabolic activity was higher in southern wetlands; microbial carbon source utilization preferences were categorized into labile organic carbon and recalcitrant carbon, and microbial carbon metabolic activity was regulated by soil total nitrogen.

Soil microorganisms play a crucial role in carbon processes within wetland ecosystems. Although existing studies have focused on mangrove ecological restoration and carbon sequestration potential [19,20,21,22], it remains unclear how soil microorganisms respond to and regulate the impact of exogenous nitrogen input on soil carbon sequestration, as well as how their carbon source utilization preferences change following the introduction of *Kandelia obovata* into mudflat wetlands. We therefore tested the hypothesis that, compared with the unplanted group, planting *Kandelia obovata* under appropriate nitrogen addition would increase soil total carbon (TC), microbial biomass carbon (MBC) and nitrogen (MBN), promote higher evenness and more stable functional traits of the microbial community, and drive the preferential utilization of carbon sources toward amino acid and lipid substrates. To test this hypothesis, this study aimed to: (1) To evaluate the responses of TC, MBC, MBN and microbial carbon source utilization preferences to nitrogen addition in the planted group and unplanted group; (2) Determine the optimal nitrogen level and elucidate the regulatory mechanism of *Kandelia obovata* in enhancing soil carbon sequestration. By addressing these questions, this study aims to clarify the mechanisms by which *Kandelia obovata* regulates soil carbon sequestration in coastal wetlands under nitrogen enrichment. The findings provide a scientific basis for improving soil carbon sequestration capacity in coastal wetlands by optimizing microbial carbon utilization strategies with *Kandelia obovata* under appropriate nitrogen addition.

## 2. Results

### 2.1. Effects of Nitrogen Addition on Soil Carbon Sequestration in the Planted Group and Unplanted Group

**Figure 1:**
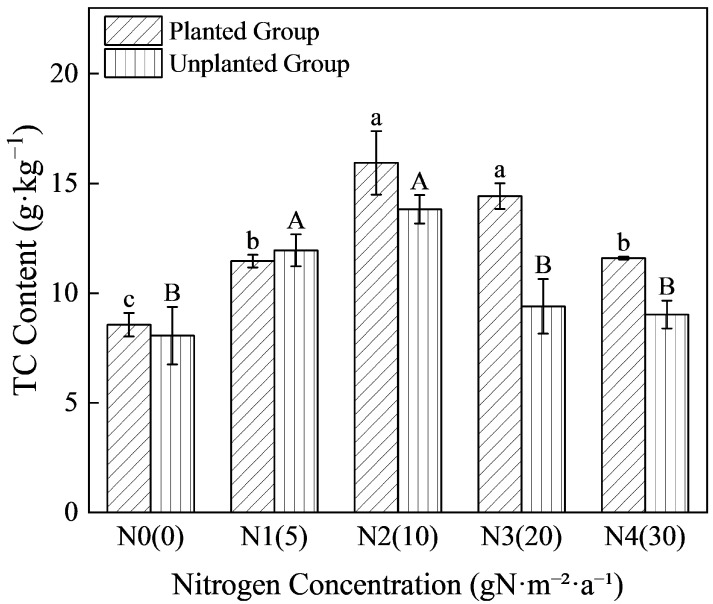
Variation Characteristics of TC contents under different nitrogen input concentrations. Different lowercase letters denote significant differences among nitrogen treatments within the planted group (*p* < 0.05); different uppercase letters denote those within the unplanted group (*p* < 0.05).

Soil total carbon (TC) was analyzed as a key indicator of carbon sequestration under nitrogen addition in the planted group and the unplanted group. As shown in Figure 1, nitrogen input significantly affected TC content in both systems (*p* < 0.05). All nitrogen-treated groups exhibited higher TC content than the N0 control, demonstrating a unimodal response (first increasing then decreasing) to increasing nitrogen concentration, with peak values at the N2 treatment (mean values of 15.94 g·kg^−1^ in the planted group and 13.82 g·kg^−1^ in the unplanted group). Except for the N1 treatment, the planted group consistently had higher TC content than the unplanted group. Specifically, compared with the unplanted group, the planted group showed increases of 6.14%, 15.34%, 53.49%, and 28.53% in the N0, N2, N3, and N4 treatments, respectively. This percentage increase also followed a unimodal pattern, peaking in the N3 treatment.

### 2.2. Effects of Nitrogen Addition on Soil Microbial Biomass in the Planted Group and Unplanted Group

**Figure 2:**
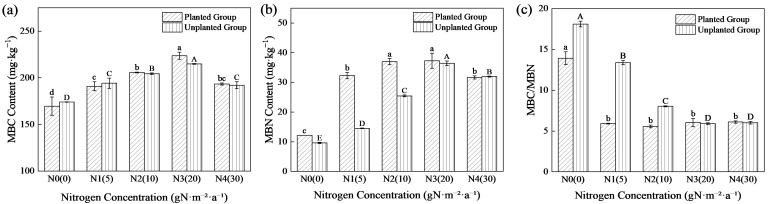
Variation Characteristics of MBC, MBN and MBC/MBN contents under different nitrogen concentrations inputs. Content of microbial biomass carbon (**a**), microbial biomass nitrogen (**b**), and MBC/MBN ratio (**c**) Different lowercase letters denote significant differences among nitrogen treatments within the planted group (*p* < 0.05); different uppercase letters denote those within the unplanted group (*p* < 0.05).

Different nitrogen addition concentrations significantly affected soil microbial biomass carbon (MBC), microbial biomass nitrogen (MBN), and the microbial biomass C:N ratio (MBC/MBN) in both systems (Figure 2, *p* < 0.05). In both systems, MBC and MBN contents increased initially and then decreased with increasing nitrogen addition, reaching the maximum under the N3 treatment, compared with the N0 treatment, the contents of MBC and MBN in the planted group under the N3 treatment increased by 31.83% and 206.24%, respectively, while those in the unplanted group increased by 23.46% and 279.03%, respectively. Compared with the unplanted group, the relative change rate of MBN in the planted group was higher than that of MBC, and the increment of MBN in the low nitrogen treatments was significantly greater than that in the medium and high nitrogen treatments. Specifically, MBN contents in the planted group under N0, N1 and N2 treatments were 26.62%, 122.4% and 45.36% higher than those in the unplanted group, respectively, which were much higher than those in other treatments. In contrast, MBC contents in the planted group under N0 and N1 treatments were 2.51% and 1.63% lower than those in the unplanted group, respectively, while MBC content in the planted group was higher than that in the unplanted group under the remaining treatments. The soil microbial biomass carbon-to-nitrogen ratio (MBC/MBN) in both groups showed a consistent trend of first decreasing significantly and then stabilizing with increasing nitrogen addition levels, but the rate of decline and stabilization threshold differed markedly between the two systems. The planted group exhibited a faster decline: MBC/MBN in the N1 treatment decreased by 57.54% compared with the N0 treatment, and remained stable from the N1 level onward, with no significant further variation with increasing nitrogen concentration. In contrast, the unplanted group showed a slower decline, with only a 26.18% reduction in MBC/MBN under N1 relative to N0, and the ratio did not stabilize until the N3 treatment. Inter-group comparison revealed that the presence of *Kandelia obovata* significantly reduced soil microbial MBC/MBN under low to moderate nitrogen addition (N0–N2).

### 2.3. Effects of Different Nitrogen Input Concentrations on Soil Microbial Carbon Source Metabolic Activity

**Figure 3:**
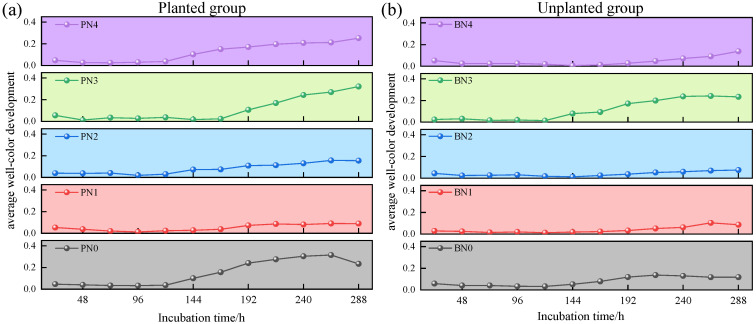
Effects of exogenous nitrogen input on average well color development of soil microorganisms. AWCD profiles of planted group (**a**) and unplanted group (**b**).

The AWCD characterizes the carbon source utilization efficiency of microbial communities. At the early stage of nitrogen application, AWCD values of soil microbial communities under different nitrogen concentrations were compared, and differences in AWCD values among different treatments were found. As shown in Figure 3, under different nitrogen input concentrations, the AWCD values in the planted group were significantly higher than those in unplanted group; specifically, under the N0, N2, and N4 treatments, the AWCD values in the planted group were approximately twice that of unplanted group, indicating that planting *Kandelia obovata* can enhance the soil microbial carbon source utilization efficiency. During the entire incubation period, AWCD values under different nitrogen input concentrations showed a trend of increasing slowly, then rising sharply, and finally tending to stabilize. From 24 h to 120 h of incubation, the difference in AWCD values between the planted group and unplanted group was not significant: both groups exhibited a relatively slow increase in AWCD values, which remained relatively low. After 120 h, the AWCD values in the planted group increased rapidly, whereas the rapid increase in AWCD values in the unplanted group occurred after 144 h. After 240 h, although the AWCD values in both the planted group and unplanted group continued to increase, their growth rates gradually decreased and eventually tended to stabilize. Under different nitrogen input concentrations, the order of AWCD values in the planted group was N3 > N0 > N4 > N2 > N1, while that in the unplanted group was N3 > N4 > N0 > N1 > N2. In the unplanted group, except for the N3 treatment, the AWCD values under other nitrogen concentration treatments changed slightly; in contrast, the AWCD values in the planted group showed significant changes under all nitrogen concentration treatments. These results suggest that the cultivation of mangrove plants (*Kandelia obovata*) promotes the microbial utilization of soil carbon sources.

### 2.4. Effects of Different Nitrogen Input Concentrations on the Utilization Characteristics of 6 Carbon Source Categories by Microbial Communities

**Figure 4:**
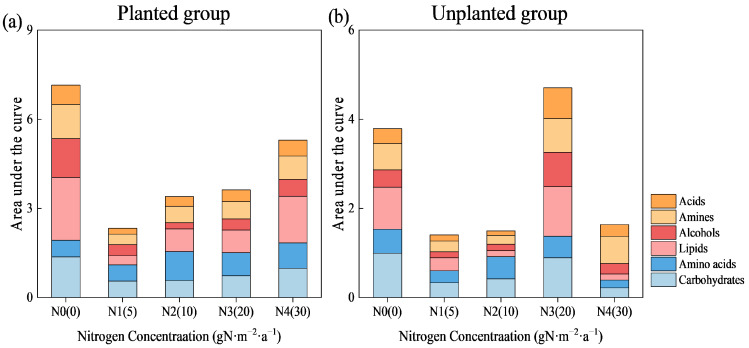
Microbial carbon source utilization activity (AUC) under different nitrogen addition levels. Carbon source utilization of the planted group (**a**) and unplanted group (**b**).

The Biolog-ECO microplate contains 31 carbon sources in total, categorized into six types: carbohydrates (7 types), amino acids (6 types), lipids (4 types), alcohols (3 types), amines (3 types), and acids (8 types). Figure 4 and Table 1 show the metabolic activity and metabolic differences in soil microorganisms after 240 h of incubation. Exogenous nitrogen input inhibited the microbial utilization of carbon sources. Under nitrogen-free conditions (N0 treatment), the microbial metabolic capacity for all carbon source categories in the planted group was higher than that in the unplanted group, with the total metabolic capacity increasing by 88.61% compared to the unplanted group. After exogenous nitrogen input, except for the N3 treatment, the carbon source utilization in the planted group was higher than that in the unplanted group under all other treatments; in the N3 treatment, only the utilization of amino acid-based carbon sources was higher in the planted group than in the unplanted group. In the planted group, except for amino acid-based carbon sources, the microbial utilization of other carbon source categories increased with the increase in nitrogen concentration, but all remained lower than that in the nitrogen-free treatment (N0). In the unplanted group, the utilization of the six carbon source categories showed a trend of first increasing and then decreasing; except for amino acid-based carbon sources, the utilization of other carbon sources reached the maximum in the N3 treatment. Specifically, the utilization of lipid-, amine-, alcohol-, and organic acid-based carbon sources in the N3 treatment was significantly higher than that in other treatments and also higher than that in the nitrogen-free treatment. Additionally, the utilization of amine-based carbon sources in the N3 and N4 treatments was higher than that in the nitrogen-free treatment.

In terms of the microbial carbon source utilization strategy in response to nitrogen concentration, the observations were as follows: (1) Under nitrogen-free conditions (N0), the unplanted group preferentially utilized carbohydrate-based carbon sources, while the planted group showed a greater tendency to utilize lipid-based carbon sources. The utilization of amino acid-based carbon sources was the lowest in the planted group, and the utilization of alcohol-based carbon sources was 235.64% higher than that in the unplanted group. (2) After exogenous nitrogen input, the soil microorganisms in the planted group showed the highest utilization rate for amino acid-based carbon sources; however, when the nitrogen concentration exceeded that of the N3 treatment, the carbon source with the highest utilization rate shifted to lipids, followed by carbohydrate-based carbon sources. With the increase in nitrogen concentration, the preferred carbon sources shifted in the order of “amino acids → lipids,” indicating a relatively stable carbon source utilization preference. In contrast, the microbial carbon source utilization strategy in the unplanted group underwent a systematic shift with increasing nitrogen concentration, with the preferred carbon sources changing in the order of “carbohydrates → amino acids → lipids → amines.” This reflects a dynamic adaptation process in which microorganisms continuously adjust their resource utilization strategies to survive and maintain ecological functions.

### 2.5. Variation Characteristics of Soil Microbial Community Diversity Indices Under Different Nitrogen Input Concentrations

**Figure 5:**
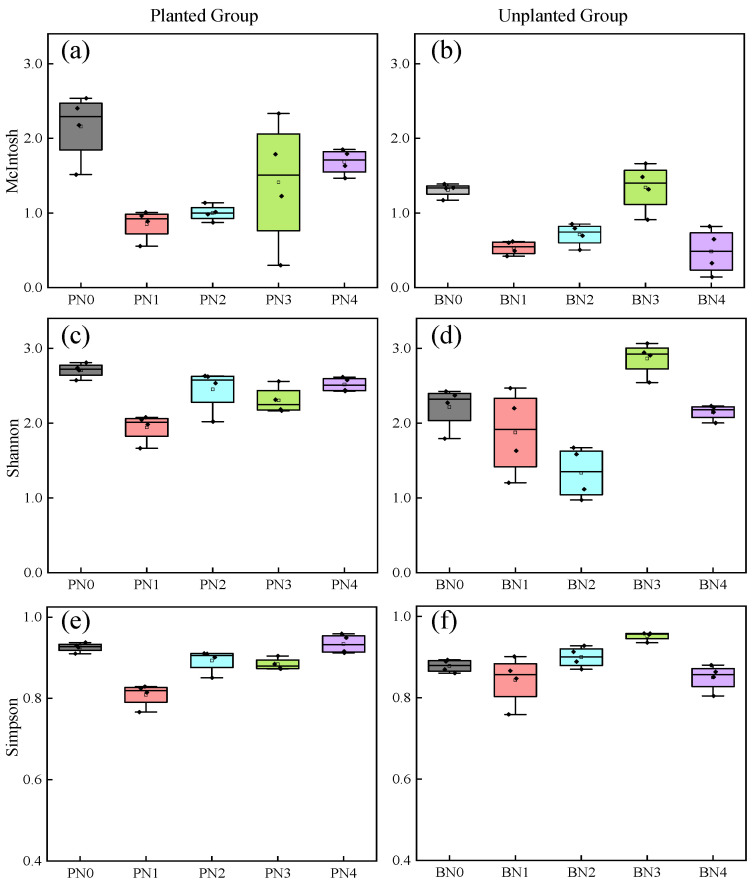
Changes in diversity indices of soil microbial communities under different nitrogen input levels. Diversity indices of McIntosh (planted group, (**a**)), McIntosh (unplanted group, (**b**)), Shannon (planted group, (**c**)), Shannon (unplanted group, (**d**)), Simpson (planted group, (**e**)), and Simpson (unplanted group, (**f**)).

Figure 5 presents the species diversity index, dominance index, and evenness index of soil microorganisms after 240 h of incubation, and the functional diversity indices of microbial communities differed across various treatment groups, indicating that the functional diversity of microbial communities varies under different nitrogen input concentrations. The results showed that under exogenous nitrogen input, the species diversity index, dominance index, and evenness index of the planted group (planted with *Kandelia obovata*) generally increased with increasing nitrogen input concentration, but were lower than those in the N0 (nitrogen-free) treatment—with the exception of the dominance index—while the unplanted group exhibited a trend of first increasing and then decreasing in these indices as nitrogen input concentration rose, with the indices reaching their maximum values in the N3 treatment (and being higher than those in the N0 treatment at this peak). A comparison of the microbial evenness index revealed that the evenness index of the planted group was higher than that of the unplanted group under all nitrogen concentration treatments. Under the N3 treatment, the microbial diversity index of the planted group was lower than that of the unplanted group, while under other nitrogen concentration treatments, it was higher than that of the unplanted group. Regarding the microbial dominance index, the planted group under the N3 treatment showed the greatest decrease compared with the unplanted group, followed by the N1 and N2 treatments; in contrast, the microbial dominance index of the planted group under the N0 and N4 treatments was higher than that of the unplanted group. Overall, a comprehensive analysis of these three microbial indices across the two systems showed that in the planted group, the N3 treatment had relatively high diversity and evenness indices alongside a relatively low dominance index, which indicates that the microbial community in this treatment had high species richness and a relatively balanced species distribution.

### 2.6. Correlation Analysis and Pathway Study of Kandelia obovata Plant–Soil–Microbe Interactions

**Figure 6:**
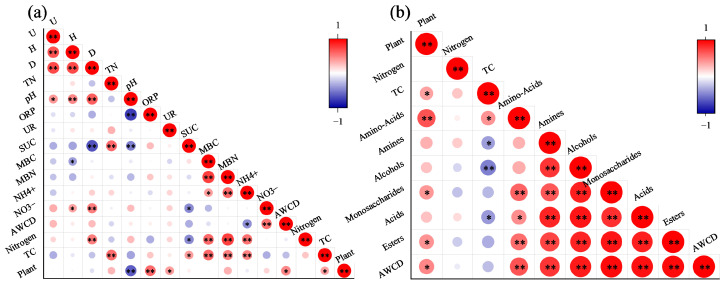
Correlation heatmap between environmental factors and carbon processes under different exogenous nitrogen input conditions. Correlations of soil physicochemical properties, microbial biomass, diversity indices and experimental factors (**a**), and carbon source utilization categories, environmental factors and overall functional activity (AWCD) (**b**). Asterisks indicate statistical significance: * *p* < 0.05, ** *p* < 0.01.

Correlation analysis was conducted between TC content and soil biochemical indices in both the planted group and the unplanted group to explore how *Kandelia obovata* and microorganisms affect soil carbon sequestration under different nitrogen input concentrations (Figure 6a). In both systems, nitrogen input showed an extremely significant positive correlation with MBC, MBN, NH_4_^+^, and the dominance index (*p* < 0.05), and a significant negative correlation with SUC (*p* < 0.05); TC content was significantly positively correlated with NH_4_^+^, SUC, MBC, MBN, and TN (*p* < 0.05), while *Kandelia obovata* showed a significantly significant positive correlation with TC, indicating that *Kandelia obovata* can promote soil total carbon accumulation, and in addition, *Kandelia obovata* also showed a significant positive correlation with ORP, UR, and AWCD, and a significant negative correlation with soil pH (*p* < 0.05).

Furthermore, analysis of the correlations between nitrogen input, *Kandelia obovata*, metabolism of different carbon sources, and carbon accumulation (Figure 6b) revealed that *Kandelia obovata* had a positive correlation with the utilization of all carbon source categories, with an extremely significant correlation with amino acid-based carbon sources and significant correlations with carbohydrate-based, lipid-based carbon sources, and AWCD, indicating that the introduction of mangrove plants is the main factor influencing the utilization of amino acid-based carbon sources and is conducive to promoting microbial metabolic activity; TC showed a significant positive correlation with amino acid-based carbon sources and negative correlations with the other five carbon source categories, suggesting that the utilization of amino acid-based carbon sources is conducive to promoting soil carbon accumulation; amino acid-based carbon sources showed an extremely significant positive correlation with carbohydrate-based, lipid-based carbon sources, and AWCD, indicating that after the introduction of mangrove plants, the promotion of amino acid-based carbon source utilization also stimulates the utilization of carbohydrate-based and lipid-based carbon sources.

**Figure 7:**
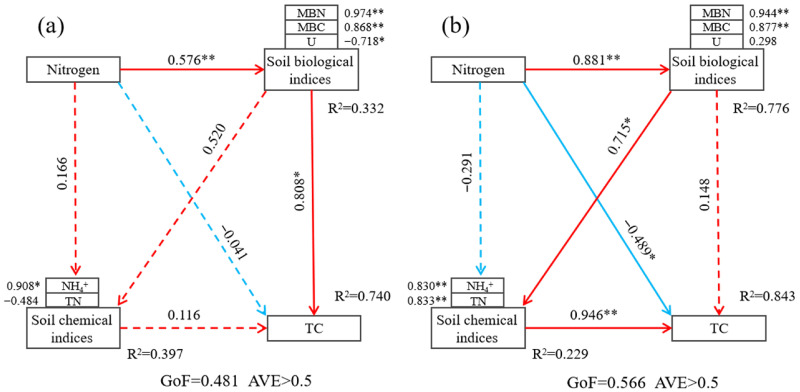
Structural equation models illustrating the direct and indirect effects of exogenous nitrogen input on soil total carbon via soil chemical and biological indices in (**a**) the planted group and (**b**) the unplanted group. Red solid/dashed lines denote positive effect pathways, while blue solid/dashed lines denote negative effect pathways. Standardized path coefficients are presented alongside the arrows, with asterisks indicating statistical significance: * *p* < 0.05, ** *p* < 0.01. *R*^2^ values represent the proportion of variance explained for each endogenous variable, and GoF (Goodness of Fit) reflects the overall model fit.

On the basis of correlation analysis, the PLS-SEM model was used to explore the pathway by which the introduction of *Kandelia obovata* affects stable carbon sequestration in bare flats under exogenous nitrogen input, with standardized path coefficients (SPC) used to represent relationships between adjacent latent variables (direct effects) or non-adjacent latent variables (indirect effects); in this study, three latent variables (exogenous nitrogen input, soil biological indices, and soil chemical indices) were constructed to explore their causal relationships with soil carbon storage, where values on the arrows and arrow thickness indicate the degree of influence and significance level, respectively. In the planted group, three latent variables explained 74.0% of the variation in soil total carbon (TC) (R^2^ = 0.740; Figure 7a). The model goodness of fit (GOF) was 0.481, and the average variance extracted (AVE) of each latent variable exceeded 0.5, indicating satisfactory model fit, reliability and validity. Structural modeling showed that exogenous nitrogen input had no significant direct effect on soil TC (*p* > 0.05), and its regulation of TC was mainly achieved through indirect pathways mediated by soil microbial biomass carbon (MBC) and microbial biomass nitrogen (MBN). The standardized path coefficient of this indirect pathway was 0.808, suggesting a strong indirect regulatory effect of exogenous nitrogen input on soil TC. In the unplanted group, the three latent variables showed higher explanatory power for soil TC variation (R^2^ = 0.843; Figure 7b). The model GOF was 0.566, and the AVE of each latent variable exceeded 0.5, indicating satisfactory model fit, reliability and validity. Unlike the planted group, exogenous nitrogen input in this group showed a dual pathway of influence on soil TC: on the one hand, exogenous nitrogen input could directly affect soil TC content (SPC = −0.489), showing a significant direct effect; on the other hand, it could further affect nitrogen-related soil chemical indices by regulating soil biological indices, ultimately exerting an indirect effect on soil TC accumulation. A comparison of the PLS-SEM results between the two systems showed that the mechanism by which exogenous nitrogen input affects soil carbon sequestration differs significantly depending on the presence or absence of *Kandelia obovata*: in the planted group, nitrogen input mainly indirectly promotes TC sequestration by increasing soil microbial diversity and biomass; in the unplanted group, however, the influence pathway of nitrogen input is more complex, involving both direct regulation of TC and indirect regulation through biological and chemical indices. This difference may reflect the potential role of mangrove plants (*Kandelia obovata*) in mediating the effects of nitrogen deposition on soil carbon cycling in coastal wetlands under our short-term laboratory simulation. It suggests that introducing *Kandelia obovata* to establish mangrove vegetation in bare flat habitats may help optimize wetland carbon sink function under nitrogen input, and provides a potential ecological regulation strategy for improving soil carbon sequestration in coastal wetlands.

## 3. Discussion

### 3.1. Effects of Nitrogen Input on Carbon Sequestration Characteristics in the Kandelia obovata-Soil System

As the preferred tree species for coastal wetland restoration and a key carrier of blue carbon sequestration, mangroves are of great significance for studying ecological adaptation mechanisms under the dual context of global high nitrogen deposition and coastal wetland restoration practices in China [2]. This study focused on *Kandelia obovata*, the core afforestation species following Spartina alterniflora removal in Fujian, and conducted targeted research in a high-nitrogen input area at the Quanzhou Bay estuary. The study found that under different nitrogen input concentrations, wetland soil microbial carbon source utilization rates entered a rapid growth phase at ~120 h, whereas the AWCD growth rate of forest soil microorganisms accelerated significantly earlier, with a rapid improvement in functional activity observed at ~24 h [23]. This may be attributed to the good aeration of forest soils, which allows aerobic microorganisms to use oxygen directly for aerobic respiration via short metabolic pathways. In contrast, the long-term waterlogging of wetlands compels microorganisms to decompose carbon sources through complex metabolic pathways.

Indoor tidal simulation showed that moderate and low rates of nitrogen addition significantly enhanced soil carbon sequestration, whereas the stimulatory effect was markedly weakened when nitrogen input reached the threshold of 20 g N (N3). These findings are consistent with those of Liu et al., who reported that nitrogen deposition promotes ecosystem carbon sequestration but leads to nitrogen saturation (20kg·ha^−1^·yr^−1^) and a blunted stimulatory effect under high nitrogen loading [24], and corroborate the results of Luo et al. from field mangrove sediment experiments [25]. Collectively, they demonstrate a dose-dependent nonlinear response of coastal wetland carbon sequestration to nitrogen input: low nitrogen input facilitates carbon sequestration, while excessive nitrogen reduces the stimulatory effect and even induces carbon loss. A comparison between our indoor simulation and field studies confirms that our indoor system realistically reflects the ecological processes of coastal wetlands under nitrogen pollution stress.

With increasing nitrogen addition, TC content tended to increase initially and then decrease, while MBC content displayed a similar unimodal pattern. Nevertheless, the peak values of TC and MBC were observed at different nitrogen concentrations, and no significant linear correlation was detected between these two parameters. This is primarily because nitrogen is a key limiting nutrient in coastal wetland ecosystems [7]: low nitrogen input alleviates ecosystem nitrogen limitation, while excessive nitrogen acts as a stress factor once the threshold is exceeded [26,27]. Soil microorganisms can acutely sense changes in exogenous nitrogen input and maintain the dynamic balance of system carbon processes by altering carbon source utilization preferences [28]. Under moderate nitrogen input, microorganisms prefer labile carbon sources for growth and reproduction; excessive nitrogen induces a shift in metabolic allocation. To mitigate nitrogen toxicity, microorganisms tend to increase the secretion of extracellular polymeric substances (EPS), reducing carbon allocation to growth and diverting more toward basal metabolism [29,30].

### 3.2. Effects of Kandelia obovata Planting on Soil Microorganisms in Coastal Wetlands

You H M et al. [20] reported that the presence of mangrove plants promotes soil microbial activity, and this study reached the same conclusion: under all nitrogen input concentrations, the AWCD values in the planted group were higher than those in the unplanted group. This indicates a close synergistic interaction between *Kandelia obovata* and soil microorganisms, which enhances microbial utilization of carbon sources [31]. As plants prioritize alleviating resource constraints in their environmental adaptation strategies, and mangroves are nitrogen-demanding species [32], their roots continuously secrete amino acids and soluble nitrogen-containing organic compounds into rhizosphere sediments, supplying abundant amino acid substrates for microorganisms. In contrast, the unplanted group lacks root-derived organic inputs, resulting in a severe scarcity of amino acid substrates [33]. Since amino acid-based carbon sources provide both carbon and nitrogen, exogenous nitrogen addition rapidly stimulates soil microorganisms to utilize such carbon sources. This not only promotes plant growth but also facilitates effective adaptation to stressful environments. Continuous exogenous nitrogen input forces microorganisms to continuously assimilate nitrogen; however, supersaturated nitrogen inhibits growth, leading to the accumulation of free ammonium/amino acids in cells, which in turn induces toxicity. Additionally, the acidic environment exerts an inhibitory effect on many enzymes involved in glycolysis and amino acid metabolic pathways [34,35]. Under high-nitrogen treatments in this study, soil microorganisms in the planted group adopted a strategy of prioritizing the utilization of lipid-based carbon sources. This may be because lipid-based carbon sources have an extremely high C/N ratio and contain almost no nitrogen in their molecular structure—when nitrogen toxicity occurs, microorganisms can avoid consuming scarce nitrogen resources by prioritizing lipid utilization, and lipid metabolism does not produce nitrogenous waste, thus preventing further nitrogen toxicity from microbial metabolites in high-nitrogen environments [36].

The microbial carbon source utilization strategy in response to nitrogen was relatively stable in the planted group, with amino acid-based and lipid-based carbon sources being the primary preferred carbon sources. In contrast, the microbial carbon source utilization in the unplanted group exhibited greater variability in response to nitrogen, undergoing a systematic shift as nitrogen concentration increased: the preferred carbon sources changed in the order of carbohydrates → amino acids → lipids → amines, reflecting a dynamic adaptation process in which microorganisms continuously adjust their resource utilization strategies to survive and maintain ecological functions. Comparatively, the ecosystem of the planted group was more stable: in addition to the stable carbon source utilization strategy, the microbial evenness index of the planted group was higher than that of the unplanted group. Under threshold nitrogen concentrations, the planted group also reduced microbial diversity and dominance to prevent excessive reproduction of dominant species, thereby promoting the uniform distribution and stability of the microbial community, regulating adaptation to the threshold effect of nitrogen input, enhancing the ecosystem’s buffering capacity against exogenous nitrogen, and exhibiting stronger adaptability-all of which are more conducive to effectively coping with global climate change. Similar conclusions have also been reported by Yang J [37] and Lyu D M et al. [38].

### 3.3. Effects of Kandelia obovata Planting on Soil Carbon Sequestration Characteristics in Coastal Wetlands

Lipid degradation products and amino acid metabolites of plant and microbial origin may interact with the positively charged hydroxyl moieties on iron and aluminum oxides via their polar functional groups through ligand exchange, electrostatic adsorption and hydrogen bonding, thereby forming mineral-associated organic carbon (MAOC) and extending the carbon turnover period to a centennial scale. This represents an important mechanism for the formation of stable carbon pools [39,40]. Additionally, during the growth of *Kandelia obovata*, plant residues and microbial residues can also bind with iron and aluminum oxides through electrostatic adsorption [41]. After utilization, lipid-based carbon sources can be converted into triglycerides stored in lipid droplets, serving as long-term carbon/energy reserves, which to a certain extent promotes carbon accumulation or conversion into storage substances [42]. In this study, the carbon source utilization strategy of soil microorganisms in the planted group was dominated by amino acid-based and lipid-based carbon sources, and the microbial utilization of carbon sources increased with rising nitrogen concentration. This microbial carbon source utilization preference in response to nitrogen in the planted group provides strong evidence for the conclusion by You et al. [20] that the stable carbon content in plant-present groups increases with nitrogen concentration after nitrogen application. K-strategist microorganisms possess the core characteristics of slow growth, high resource use efficiency, strong stress resistance, and preference for complex and recalcitrant substrates, which is consistent with the microbial preference for amino acids and lipids observed in this study. Their stable metabolic pattern effectively reduces carbon loss and continuously supplies precursors for the formation of stable carbon pools [43,44].

Furthermore, this study found that the introduction of mangrove plants is beneficial for enhancing the carbon sequestration effect of bare flats: *Kandelia obovata* showed a significant positive correlation with soil TC content (Figure 6a), directly indicating that the introduction of mangrove plants can promote total carbon accumulation in bare flat soils. The N3 treatment represents a critical threshold for soil total carbon (TC) accumulation; the planted group exhibited the most significant TC increment under this threshold, while exceeding the N3 threshold would diminish the TC enhancement effect. TC was significantly positively correlated with amino acid-based carbon sources; the utilization of amino acid-based carbon sources is conducive to promoting soil carbon accumulation. Specifically, the utilization of amino acid-based carbon sources in the N3 treatment group was higher than that in the unplanted group, while the utilization of other carbon sources was lower. In addition, the planted group under the N3 treatment had the greatest increase in MBC compared with the unplanted group—these factors collectively rendered the N3 treatment a critical threshold for TC accumulation, with TC increments declining once the N3 threshold was surpassed. The PLS-SEM model showed that after nitrogen application, in the planted group, exogenous nitrogen mainly regulated carbon sequestration indirectly by influencing microbial-related biological indicators such as microbial biomass (MBC, MBN) (with a standardized path coefficient of 0.808), while its direct effect on TC content was not significant. In contrast, in the unplanted group, nitrogen input not only dominated carbon dynamics indirectly through soil biochemical indicators but also had a significant direct effect on TC content. This may indicate that the presence of *Kandelia obovata* could reshape the regulatory network of nitrogen-carbon interactions under our experimental conditions, and that microorganisms might act as important drivers in carbon cycling processes.

## 4. Materials and Methods

### 4.1. Experimental Setup for Tidal Simulation

**Figure 8:**
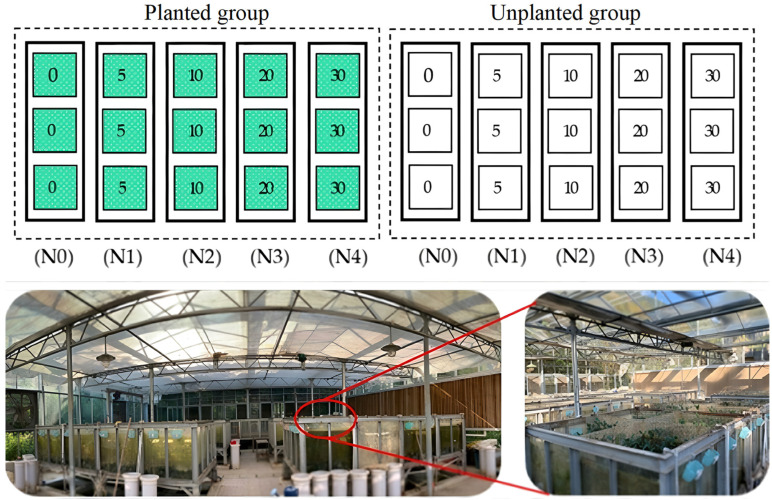
Plot Overview Schematic Diagram.

The experiment was conducted in May 2019 at the Tidal Simulation Laboratory of Fujian Academy of Forestry. Two simulated systems were established (as shown in Figure 8), including the planted group and the unplanted group as the control. According to Huang [45], the average total nitrogen deposition in Xiamen, which is adjacent to Quanzhou Bay, was 1.89 g N·m^−2^·a^−1^. We therefore adopted 2 g N·m^−2^·a^−1^ as the baseline nitrogen deposition level, and set up five nitrogen addition treatments corresponding to 0, 2.5, 5, 10, and 15 times the baseline value, with three replicates per treatment. The gradient was designated as: N0 (0 g N·m^−2^·a^−1^), N1 (5 g N·m^−2^·a^−1^), N2 (10 g N·m^−2^·a^−1^), N3 (20 g N·m^−2^·a^−1^), and N4 (30 g N·m^−2^·a^−1^). The automated tidal simulation system consisted of an upper simulation tank (1 m × 1 m × 1 m) and a lower water storage tank, connected by a water pump and drainage valve for tidal circulation. The substrate in the simulation tank was collected from the 0–30 cm surface soil of the Quanzhou Bay Estuarine Wetland (basic soil properties, Table 2), and seawater with a salinity of 12‰ was used in the tanks. According to the semi-diurnal tidal regime dominated in the *Kandelia obovata* distribution area of Quanzhou Bay, the tidal cycle was set to 12 h, with two flooding events per day (submerged flooding, 4 h per flooding). The entire system was pre-operated for one month to stabilize environmental conditions before the formal incubation. Healthy one-year-old *Kandelia obovata* seedlings with uniform size were collected from Quanzhou Bay Estuarine Wetland and transplanted into the simulation tanks (48 seedlings per tank), while no seedlings were planted in the unplanted group. Nitrogen was applied monthly as NH_4_NO_3_ solution for a total of six applications during the experiment [20].

### 4.2. Biolog ECO Plate Analysis

Soil microbial functional diversity and carbon source utilization potential were determined using Biolog ECO microplates (Wuhan Kehaojia Biotechnology Co., Ltd., Wuhan, China). Each plate contained 31 ecologically typical carbon sources and one blank control, with three technical replicates for each substrate, totaling 96 wells. The 31 carbon sources were divided into six categories: amines, amino acids, carbohydrates, acids, lipids, and alcohols.

Ten grams of fresh soil was added to a triangular flask containing 90 mL of sterilized 0.85% NaCl solution, shaken at 25 °C and 200 r/min for 30 min, and serially diluted to 10^−3^ g/mL. Then, 125 μL of the diluted soil suspension was inoculated into each well. After inoculation, the microplates were incubated continuously at 25 °C in the dark, and absorbance was measured at 590 nm using a microplate reader [46]. The initial absorbance was measured immediately after inoculation, followed by measurements every 24 h for a total of 288 h. The absorbance values of each carbon source well were corrected using the blank control well, and the area under the absorbance–time curve was calculated according to the method of Hackett & Griffiths (1997) to represent the total microbial substrate utilization activity [47].

To eliminate the interference of inoculum density and differences in microbial basal metabolism on color development, all data were standardized to the physiological stage at which the average well color development (AWCD) = 0.09 to enable comparable analysis of community-level physiological profiles among different samples. Specifically, the absorbance values of each sample at AWCD = 0.09 were extracted to calculate the relative absorbance of each well. The relative absorbance values were standardized to a total absorbance of one per plate, and the mean absorbance values were then calculated for each of the six carbon source categories for subsequent analysis [48]. This approach characterizes potential metabolic capacity under standardized laboratory conditions rather than in situ carbon cycling rates.

### 4.3. Sample Collection and Determination

Soil samples were collected on the 10th day after the first nitrogen application. Following the five-point sampling method, surface soil samples (0–10 cm depth) were collected from each culture tank 30 min before high tide and 30 min after low tide. After removing impurities from the soil samples, the samples were placed in self-sealing bags, stored in an insulated container, and transported back to the laboratory. Subsequently, the soil samples were mixed using the quartering method and passed through a 2-mm sieve. They were then divided into two portions for different analyses: One portion was stored at 4 °C to determine the following indicators: soil microbial carbon source metabolic activity (represented by average well color development, AWCD), soil pH, ammonium nitrogen (NH_4_^+^-N), nitrate nitrogen (NO_3_^−^-N), oxidation-reduction potential (ORP), microbial biomass carbon (MBC), and microbial biomass nitrogen (MBN). The AWCD was measured using the Biolog-ECO microplate method. The other portion was air-dried and ground using a ball mill to determine soil total carbon (TC), total nitrogen (TN), urease (UR) activity, and sucrase (SUC) activity.

Soil pH, MBC, MBN, NH_4_^+^-N, NO_3_^−^-N, UR activity, and SUC activity were all determined using conventional soil agrochemical analysis methods [49]. Soil TC and TN were determined using an elemental analyzer.

The overall capacity of soil microbial communities to utilize carbon sources is represented by the AWCD [50]. Microbial diversity is characterized using three indices: the Shannon diversity index (H) [51], Simpson dominance index (D) [52], and McIntosh evenness index (U). The calculation formula is as follows:

(1) $$AWCD=\frac{\sum \left(C_{i}-R\right)}{n}$$

(2) $$H=-\sum P_{i}\ln P_{i},\;P_{i}=\frac{\left(C_{i}-R\right)}{\sum (C_{i}-R)}$$

(3) $$D=1-\sum P_{i}^{2}$$

(4) $$U=\sqrt{\left(\sum n_{i}^{2}\right)},\;n_{i}=C_{i}-R$$

where *C_i_* is the absorbance value of each carbon source well; *R* is the absorbance value of the control well; *n* is the number of carbon sources (specifically, *n* = 31, consistent with the 31 carbon sources in the Biolog-ECO microplate).

### 4.4. Data Analysis

Data analysis was performed using the following software: Microsoft Excel 2019 for data preprocessing; SPSS 26.0 for statistical analyses, including significance tests (LSD, *p* < 0.05), data standardization, and Pearson correlation analysis; SmartPLS 4.0 for building a Partial Least Squares Structural Equation Model (PLS-SEM); and Origin 2021 for plotting all graphs.

## 5. Conclusions

This study conducted an indoor tidal simulation control experiment to systematically explore the response mechanisms and adaptability of coastal mangrove ecosystems to nitrogen deposition. By determining soil biochemical indices, microbial carbon source utilization efficiency, and microbial diversity, the study concluded that under exogenous nitrogen input, *Kandelia obovata* reshaped the microbial carbon metabolic patterns and community structures by providing carbon sources and modifying the microenvironment; in turn, microorganisms exerted a feedback effect on the *Kandelia obovata*-driven carbon sequestration process by enhancing carbon source utilization efficiency and biomass accumulation. After exogenous nitrogen input, soil microorganisms in the planted group enhanced their adaptability to nitrogen by reducing dominance and increasing evenness, and adapted to environmental changes by adjusting and stabilizing their carbon source utilization strategies. Notably, the stable utilization of amino acid-based and lipid-based carbon sources is a key mechanism for the stable carbon sequestration of mangrove plants. Overall, this study deepens the understanding of the response mechanisms of coastal wetland carbon cycling to global changes and provides an important reference basis for nitrogen pollution prevention and control in coastal zones, estimation of mangrove carbon sink potential, and adaptive management of mangrove ecosystems.

## Figures and Tables

**Table 1:** Change rate of carbon source metabolism in the planted group compared with unplanted group (%).

Carbon Sources	N0	N1	N2	N3	N4
Carbohydrates	37.60	66.57	37.65	−18.05	353.70
Amino acids	4.46	105.20	93.59	60.33	395.95
Lipids	124.15	4.81	466.18	−31.87	1034.06
Alcohols	235.64	172.26	46.48	−50.52	146.75
Amines	92.76	46.89	175.88	−23.09	27.97
Acids	96.12	44.20	220.39	−43.27	106.54
Total metabolic activity	88.61	65.91	127.07	−23.07	224.37

**Table 2:** Soil physicochemical properties of the simulated experimental soil.

Volumetric Weight (g/cm^3^)	TC Content (%)	TN Content (%)	NH_4_^+^-N (mg/kg)	NH_3_^−^-N (mg/kg)
62.75 ± 0.03	1.89 ± 0.13	0.13 ± 0.0025	3.24 ± 0.34	2.71 ± 0.12

Note: The data in the table are mean I standard deviation.

## Data Availability

The data are contained within this article.
